# The Notification of Venereal Diseases

**Published:** 1916-10-28

**Authors:** 


### THE NOTIFICATION OF VENEREAL DISEASES.

During the few months which have elapsed since the Report of the Royal Commission on Venereal Diseases was issued there has been a steadily increasing public interest in this grave and intricate question, and a vast deal of steady spade-work has been accomplished in preparing the ground for legislative and other actions. Notably the Local Government Board, under the wise direction of the Right Honourable Mr. Walter Long, has done most valuable work in keeping local authorities alive to their duties and their opportunities of carrying out many of the recommendations of the Royal Commission. Many local schemes are being prepared, and some have even got to the stage of completion. Many hospitals, great and small, are engaged in plans of co-operation by providing facilities for treatment in connection with these schemes. Many valuable articles have appeared in the press, daily and weekly, Metropolitan and provincial; the press, in fact, is doing its share in that process of educating the public over a question which has for much too long been shelved and shunned for insufficient reason. Perhaps of all the newspapers the *Morning Post* deserves most credit for the way it has handled this topic; but others have also done well. Over and beyond all this, the National Council for Combating Venereal Diseases has been most active in arranging meetings and in propaganda work of all kinds. Beyond all doubt, the British public is waking up to the urgency of tackling a source of national weakness and decay which our statesmen, if we have any, ought certainly to have brought before public notice years ago. It is reasonably certain that the measures already taken and to be taken will make a very perceptible difference to the prevalence of these diseases, and will have the further effect of rousing the public conscience to further effort and more drastic legislative action.

This, however, is unfortunately not enough for certain hot-headed people. Not content with measures incalculably in advance of anything hitherto attempted, they want to concentrate on a proposal for compulsory notification which the Royal Commission expressly deprecated for the present. They have issued a manifesto which is virtually an attempt to force the hands of the Local Government Board and of the National Council for Combating Venereal Diseases over this question. The manifesto is intemperate in tone and in language; and it takes several assumptions for granted which are either highly disputable or at least insufficiently supported by evidence. The manifesto is signed by several social workers of considerable experience, though not many of them can be regarded as competent authorities on the highly complicated subject of venereal disease. Some of them have already long been distinguished for an impetuosity and an exuberance of the kinds which defeat their own ends more often than not; and the attitude of their manifesto is one of scarcely disguised hostility to the National Council and to the Local Government Board's line of policy. The former of these two bodies has lost no time in issuing a refutation of the "Social Workers'" manifesto. To these hot-heads necessary caution appears as timidity; but at the risk of incurring their contempt, we feel obliged to deprecate very strongly indeed their rash attempt to force the pace. It is a thousand pities that, just as real progress is well in prospect, and the co-operation of all thoughtful citizens is required to build its foundations securely, the least semblance of disunion should appear. If there is one thing more calculated than another to make the public lukewarm in the whole matter, it is wrangling and squabbling amongst those who pose as its teachers and mentors. Even if the "Social Workers" were incontestably right in their views—which is, after all, a matter of opinion—the action they have taken is not calculated to advance those views. This particular piece of social progress is one in which a decent unanimity and the support of public opinion are above all other things the most essential; there is every chance of securing both if only the fanatics can be restrained.

